# A new measurement method for spine reposition sense

**DOI:** 10.1186/1743-0003-5-9

**Published:** 2008-03-26

**Authors:** Cheryl M Petersen, Chris L Zimmermann, Steven Cope, Mary Ellen Bulow, Erinn Ewers-Panveno

**Affiliations:** 1 Concordia University Wisconsin, 12800 North Lake Shore Drive, Mequon, WI, 53097, USA; 2 Athletico, 1500 Waukegan Road, Suite 250, Glenview, Illinois, 60025, USA; 3 Core Control LLC, Chicago, Illinois, 60610, USA

## Abstract

**Background:**

A cost effective tool for the measurement of trunk reposition sense is needed clinically. This study evaluates the reliability and validity of a new clinical spine reposition sense device.

**Methods:**

The first part of this three part investigation included 45 asymptomatic subjects examined in the first 20 repeated trials portion assessing spine reposition sense. The second portion, test-retest, examined 57 asymptomatic subjects. Initial testing consisted of subjects sitting on the device and performing 20 trials of a self-determined 2/3 trunk flexion position. The second portion of the study involved 7 trials of trunk flexion performed twice. The angular position for each trial was calculated and the mean reposition error from the initial 2/3 position was determined. For the third portion, the new device was compared to the Skill Technologies 6D (ST6D) Imperial Motion Capture and Analysis System.

**Results:**

ICC (3,1) for trials 4–7 was 0.79 and 0.76 for time one and time two, respectively and the test-retest ICC (3,k) was 0.38. Due to the poor test-retest ICC, the Bland Altman method was used to compare test and retest absolute errors. Most measurement differences were small and fell within the 95% confidence interval. Comparable measures between the two methods were found using the Bland Altman method to compare the reposition sense device to the ST6D system.

**Conclusion:**

The device may be a cost effective clinical technique for sagittal trunk reposition sense measurement.

## Background

Proprioception describes those sensations generated within the body which contribute to an awareness of the relative orientation of body parts, both at rest and in motion [1]. The proprioceptive system is dependent upon simultaneous activity in a number of types of mechanoreceptor afferent neurons. Mechanoreceptors provide information for reflex regulation of muscle tone, for awareness of position sense and movement sense [2] and have been isolated in most spinal tissues [3-10].

Afferent information is processed in the CNS both at a subconscious and conscious level. The conscious component of proprioception can be measured through tests designed to examine either position sense (awareness of the relative orientation of body parts in space) or movement sense (detection of movement and acceleration) [1,11]. This investigation evaluated the conscious position sense aspect of trunk proprioception.

Proprioception training has been suggested as an important aspect of treatment intervention in low back pain rehabilitation especially over the last fifteen years. The present literature on spine proprioception rehabilitation involves primarily exercise dealing with balance, posture and stabilization. However, a specific rehabilitation program to improve spine proprioception has not been established. Ashton-Miller et al. [12] asks an important basic question: can exercise even improve proprioception? Little evidence supports the assumption that targeted exercise improves proprioception. The evidence for training to change the number of peripheral receptors is lacking. But sensory input (proprioception) processed by the central nervous system, can be modified with training [12-16].

Proprioception is considered essential for the control of human movement and can be important in diagnosing motor control impairment [13,14,17-19]. Patients with low back pain (LBP) present with both altered motor control and impaired spinal reposition sense [20-23]. Impaired motor control findings with low back pain include balance impairment [24-27], longer reaction times and decreased psychomotor speed [25,28-31], changes in trunk feed-forward control (transversus abdominus) [28,32-34] and (loss of muscular stabilization cross sectional area loss of the multifidus) [35-37]. Several studies [20,23,38-41] have compared subjects with low back pain to control subjects using various techniques. All but two of these studies [39,40] found significantly decreased reposition sense error in the subjects with low back pain compared to controls. The two studies [39,40] finding no differences compared findings between these two separate studies using the same methodology.

There are many proposed causes of low back pain but none specifically deal with documented changes in proprioception. Studies dealing with delayed trunk feed forward control [28,29,32,33] have not measured proprioception. Feed forward control of the transversus abdomnis has been delayed with both upper and lower extremity movements in subjects with low back pain compared to controls [29,32]. Delays in trunk feed forward control in the multifidus and erector spinae with expected upper extremity loading with no trunk support have been found in subjects with low back pain compared to controls [28]. Could there be an association between the decreased reposition sense that has been found in subjects with low back pain and these changes in motor control? Proprioception must be measured in studies like these to determine if there is an association between impaired motor control and proprioception involvement.

Previous descriptive studies evaluating subjects with and without low back pain have investigated proprioception in the cervical spine [19,42-44], lumbar spine [20,39-41,45-48] thoracolumbar spine [1,11,38,49], and the trunk as a whole [50,51]. These studies have established a range of trunk absolute repositioning errors associated with pelvic tilting and movements into flexion, side flexion and rotation. The reported range of absolute repositioning errors for flexion of the trunk as a whole is 1.67 – 7.1° [1,11,38,49]. Previous studies have also used repeated trials ranging from 3 to 20, 3 [41,49], 4 [47], 5 [48,50], 10 [38] and 20 [51] trials. Unfortunately the investigations using 10 or more trials have not determined if there was any change in error with a greater number of trials.

Studies have investigated the effect of muscle or mental fatigue on reposition sense in the trunk and peripheral joints utilizing computerized motion analysis devices [48,52-61]. In the spine, error values increased 1.0°–1.75° post-fatigue [48]; at the shoulder, error values increased 0.4° [53] and 2.0° [61] post-fatigue; and at the knee, error values increased 1.07° [60] and from 0.7 – 1.24° [55] post fatigue. These findings suggest that reposition sense worsens with fatigue. The potential impact of fatigue is therefore a concern when developing reposition sense test protocol.

Three spine reposition sense methods have been identified in the literature, the 3SPACE (Polhemus Navigation Sciences Division), a version of the Skill Technologies System which was used in this study, the Lumbar Motion Monitor (LMM, Chattanooga Corporation) and a piezoresistive amplified and temperature compensated accelerometer. The Skill Technologies 6D (ST6D) Imperial Motion Capture and Analysis System (Advanced Motion Measurement, LLC; 1202 E. Maryland Avenue, Suite 1G; Phoenix, AZ 85014), a form of the 3SPACE system, is an integrated magnetic tracking system using motion capture boards, a keyboard, a color monitor, one transmitter and motion capture receivers (targets). Real time position and orientation with six degrees of freedom can be produced from the motion capture receivers. A 2-inch cube transmits an electromagnetic signal that is received by sensors attached to specific parts of the body. The sensor is wired to a dedicated computer and sampled at a rate of 120 Hz. Information is stored for later viewing, data reduction, and analysis. The electromagnetic tracking system used by ST6D has <1 mm error in translation and <1° error in rotation [62]. Lam et al. [39] used the system (sensors placed at T10 and S2 spinous processes) and indicated resolution accuracy less than 0.1 degrees about the x, y and z axes for angular motion. Errors found using the system have been repeatable both between and within testing days [1]. Lumbar range of motion average values from the system compare well with values from biplanar radiography [63]. The voltage root-mean-square (vrms) (0.15 degrees), given as the angular accuracy of the system by the manufacturer, will be influenced by the distance between the sensors and the source. Swinkels & Dolan [11] found that accuracy declined in the sagittal plane from 0.29 degrees vrms when the sensors relative to the source operate at 20 cm, reaching 0.62 degrees vrms when the range increases to 81 cm. The coronal plane equivalent values are 0.72 and 0.96 degrees. The ST6D system was used within these parameters during part three of the current investigation.

All three of the above methods can accurately measure reposition sense. The accelerometer and LMM have produced even better measurements than a video-motion evaluation system considered the gold standard [64,65]. Total vrms error with the 3SPACE is less than 0.2 degrees in measuring angles. Lumbar range of motion measurements are comparable to radiographs using 3SPACE [63]. Single plane motion can only be evaluated with the accelerometer while the LMM and 3SPACE provide measurements in all three planes. Consideration of metal within the environment becomes important with the use of 3SPACE. From these positive findings, potentially any of these three devices could provide clinical measurement techniques. Despite the higher costs of either the LMM or the 3SPACE compared to the accelerometer, these costs, relative to other medical equipment, may not be extreme. The reasoning for the lack of clinical incorporation of these methodologies relates more to their ease of use and the time required to complete a measurement procedure. Environmental set-up regarding metal constraints would also be a concern with 3SPACE (Skill Technologies). Due to greater cost, increased time and less ease of use of these devices, a need for a clinical measurement tool for proprioception seemed apparent. So this new spine reposition sense device for measurements within the sagittal plane was developed.

Patients with low back pain are often treated over periods lasting several weeks in physical therapy. Insight into the test-retest reliability of this new device's ability to measure sagittal plane spinal reposition sense is essential for better understanding of the psychometric properties of the device. The use of healthy adults allows the characterization of any normal variation that could occur without the confounding effects of change that may occur within a patient population.

The goals of this study were to 1) determine the number of average trials required to produce the best reposition sense reliability (portion 1), 2) evaluate test-retest reliability of the device in measuring reposition sense error (portion 2), and 3) validate the new device against a "gold standard" (portion 3).

## Methods

### Subjects

Subjects were recruited on a volunteer basis from 2 university campuses, 45 subjects for portion 1 and 57 subjects for portion 2. Subjects who agreed to participate completed a medical questionnaire and the Oswestry Low Back Pain Questionnaire for inclusion/exclusion purposes. Entrance criteria included ≤ 5% score on the Oswestry Low Back Pain Questionnaire, a lower age limit of 18 years, set to target subjects with a fully developed proprioceptive system [12] and an upper age limit of 40 years, in an attempt to reduce the effect of age-related changes in position sense [66-69]. Exclusion criteria are presented in Table 1. Forty-five (portion 1) and 57 (portion 2) asymptomatic subjects, between the ages of 18 to 40, met the inclusion criteria and were tested. Descriptive statistics for the subjects are presented in Table 2. Informed consent was obtained from all subjects, subjected to IRB approval. Two subjects were excluded from portion 1 because data were verbalized with one subject which may have biased performance, and another subject was unable to focus on the task for the half-hour test duration.

**Table 1:** Exclusion Criteria (by self-report)

Oswestry back pain scores of greater than or equal to 5%
Balance, coordination, or stabilization therapy within the last six months
Excessive use of pain medication, drugs, or alcohol
Ligamentous injury to the hips, pelvis, or spine
Spinal surgery
Balance disorders secondary to: active or recent ear infections, vestibular disorders, trauma to the vestibular canals, or orthostatic hypotension
Neurologic disorders including: multiple sclerosis (MS), cerebral vascular accident (CVA), spinal cord injury, neuropathies, and myopathies
Diseases of the spine including: osteoporosis, instability, fractures, rheumatoid arthritis (RA), degenerative disc disease (DDD), and spondylolisthesis

**Table 2:** Descriptive Statistics for Subject Characteristics

	**Repeated Trials**	**Test-Retest**
**Number**	45	57
**Age**		
(Mean ± SD)	25.6 ± 4.2	22.2 ± 3.8
**Sex Ratio**		
Male:Female	8 : 37 (21.6%)	13 : 44 (29.5%)
**Height **(cm)		
(Mean ± SD) Female, Male	167.1 ± 7.1, 179.8 ± 8.6	167.0 ± 6.5, 181.0 ± 6.2
**Weight **(kg)		
(Mean ± SD) Female, Male	58.8 ± 8.6, 86.1 ± 13.9	66.4 ± 11.3, 87.3 ± 16.7

### Equipment

**Figure 1:**
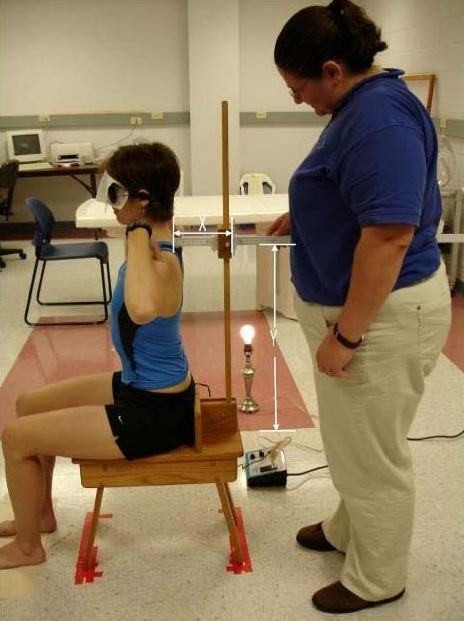
The new measurement method: X and Y coordinates are measured and used in a trigonometric calculation to determine the starting angle. An individual is shown seated in the upright starting posture; during the study, all subjects were blindfolded throughout testing.

**Figure 2:**
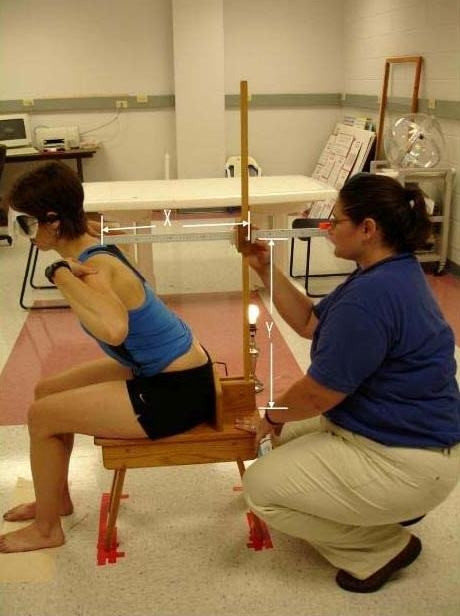
The new measurement method: The X and Y coordinates are shown above with an individual in a position 2/3 of full flexion; during the study, all subjects were blindfolded throughout testing.

The new device consists of two meter sticks and a sliding mechanism (Figures 1 and 2). One meter stick is positioned vertically and the second meter stick extends perpendicular to the vertical meter stick. The horizontal meter stick has a level attached and the vertical meter stick is perpendicular to a leveled wooden stool, upon which the subject sits. A flat piece of wood is bolted to the stool for each subject to place their sacrum against for positioning in the upright starting position. Vertical measurement is taken through an opening within the sliding mechanism and the horizontal measurement is taken from the front of the sliding mechanism, measuring the distance from the vertical meter stick to a point over the spine. The sliding mechanism allows for measurement of a wide range of subject heights and sagittal trunk motions. Leveling the entire device ensures 90° angles, enabling the use of a trigonometric equation in measuring trunk orientation and position. The measurement resolution of the new device was determined to be 0.17° (+ or -1 mm in X and Y).

### Protocol

Subjects in portion 1 and portion 2 were instructed before testing not to perform any unaccustomed strenuous physical activity for 24 hours before testing and to not eat or drink two hours prior to testing to minimize cutaneous input from a distended abdomen [40]. Testing occurred in a single session that lasted 30 minutes or less for each subject and for the test-retest portion (portion 2), subjects were seen 1 week apart within 2 hours of the previous testing time. During testing, visual input was eliminated by blindfolding the subjects and auditory input was limited by keeping the room silent [1,11,22,40,41]. Cutaneous input was minimized by instructing females to wear a halter top or sports bra and males were asked to remove their shirts for testing [22]. In addition, subjects were asked to sit upright on their ischial tuberosities and place their fingertips on their ipsilateral shoulder to limit cutaneous cues.

All subjects were asked if they were experiencing any pain the day of testing to confirm that no changes had occurred since the initial questionnaires were completed. The subjects were then palpated in sitting by examiner one and a line was marked with a pen on the top of the C7 spinous process. If measurements in forward bending could not be taken from the C7 spinous process secondary to spinal kyphosis and/or musculature, the mark was then redrawn at T4. The subsequent test-retest study used the T4 level in all 57 subjects.

Examiner two read a set of standardized instructions to each subject. Subjects were instructed that the upright starting posture included sitting up straight with their ischial tuberosities touching the stool, feet shoulder width apart and fingertips touching their ipsilateral shoulder (Figure 1). Subjects were instructed to keep their ischial tuberosities touching the stool and not to slide forward from the wood piece attached to the stool. Then, subjects were told they would be asked to bend their trunk forward, keeping a neutral neck position to both an end-range trunk flexion position, and to a position 2/3 of their full trunk flexion (Figure 2). They were instructed that full trunk flexion was the point before feeling their sacrum leave the wood piece. The subject was then instructed to estimate 2/3 of that full trunk flexion position (initial 2/3 position). Subjects were instructed to remember the initial 2/3 position in order to perform repositioning accurately throughout the 20 trials for the repeated trials portion (portion 1) and for the 7 trials for the test-retest portion (portion 2).

The measurement procedure was standardized and completed by examiner two. The X and Y coordinates were recorded for the following positions: initial position, (Figure 1) full trunk flexion position and the estimated 2/3 position (Figure 2). The subject was allowed to rest 10 seconds between each trial. Examiner two consistently measured using the line across the top of the spinous process. Examiner one wrote the data on a sheet of paper for all sets of data taken. The data were subsequently entered into an Office '97 Microsoft Excel spreadsheet designed for the study. Examiner one did not perform any measurements. The data were not verbalized to ensure the subject did not adjust their performance based on examiner verbal report of position values.

#### Portion 3: Skill Technologies ST6D compared to the new Spine Reposition Sense Device (SRSD)

In order to validate the new device, the Skill Technologies 6D (ST6D) Imperial Motion Capture and Analysis System was used as the gold standard using two methods. In the first method, a ST6D receiver was placed on the end of the horizontal meter stick and moved between 35 and 70 cm vertically and between 25 and 70 cm horizontally in 5 mm increments. These values reflect the maximum vertical and horizontal measures obtained when evaluating trunk reposition sense in 45 pilot asymptomatic subjects (+ and – 5 mm). Concurrent displacement readings from the new device and ST6D were used to calculate angles. In the second method, a single subject performed 50 trials throughout the measurement space. Calculations using the displacement data from ST6D and the new SRSD were used to determine trunk position.

### Data analysis

Calculation of the angle the trunk assumed at the 2/3 trunk flexion position was computed for each trial, using the trigonometric equation, theta = tan^-1 ^X/Y. Reposition error was calculated for trials 1–20 (repeated trials portion 1) and for trials 1–7 (test-retest portion 2) as the difference between each trial's 2/3 angle position and the initial 2/3 trial. Mean absolute error was determined for each trial as the average of the absolute value of the reposition sense error across subjects. Mean absolute reposition error (mean ARE) for each subject was calculated as the average of the sum of the reposition angle errors across trials.

#### Portion 1: Impact of repeated trials

**Figure 3:**
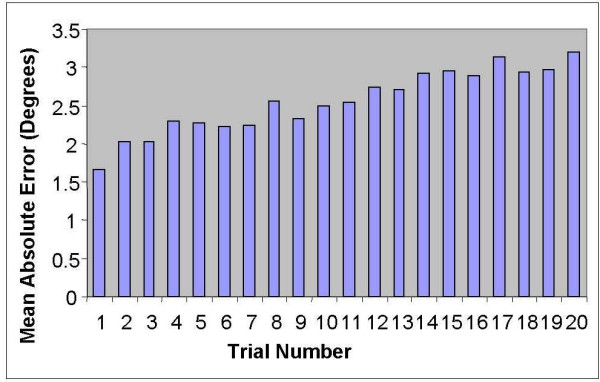
Mean reposition error from the target 2/3 position (by trial) for the 45 asymptomatic subjects with the horizontal axis representing trials 1–20 and the vertical axis representing mean reposition error in degrees. Each bar shows the mean reposition error for all the subjects tested (N = 45) for that trial.

The observation of the performance of trunk reposition sense over 20 trials was used to determine the number of trials needed for practice and the number of trials that produced the best reproducible score. A graphical analysis of the subject's 20 repeated trials of absolute reposition sense error was used to assess changes in error over trials (Figure 3). Error was noted to stabilize during trials 4–7 and increase after 7 trials.

To determine whether the graphical analysis suggesting trials 1–7 as the optimum number of trials was correct, linear regression analysis was used. Reposition sense error for all 20 trials was broken into subgroups of four trials to determine the group with the most consistent error. These subgroups were analyzed using SPSS 13.0 linear regression. The β coefficient closest to 0 as well as the magnitude of the mean absolute error of the group of 4 trials was used to determine the optimum number of trials to perform. The group of trials with the β coefficient closet to 0 and the smallest magnitude of mean absolute error were identified as being optimal.

#### Portion 2: Test-retest reliability

A paired samples t-test was used to compare time 1 to time 2 for the 7 trials with 95% confidence intervals. Calculation of ICC (3,1) for all combinations of the first 7 trials (using a minimum of two and up to seven trials) was performed using SPSS 13.0 to find the highest ICC value within these combinations for time one and time two in the test-retest portion [70,71]. Trials 4–7 produced the best results. The mean value of trials 4–7 trials for trial one and trial two was computed to be used then in an ICC (3, k) for test-retest comparison. The standard error of measurement (SEM) was calculated. A Bland-Altman plot was used to compare absolute error findings for time one versus time two for the test-retest portion [72].

#### Portion 3: Validity

Using the displacement measurements to compute angular measures from the ST6D system and from the new SRSD, an ICC (2,1) was computed. The angular difference between the ST6D and the SRSD for one subject was plotted against the mean of the two techniques using the Bland Altman method [72]. By comparing the difference between the paired measurements, the only source of variability then should be the measurement error.

## Results

### Portion1: Repeated trials

Descriptive data for time one and time two of the test-retest portion of the study can be found in Table 3. A true two-thirds position of full flexion, either at time one or time two, was achieved by the subjects. The percentage of full flexion was 66.5% and 67.6% at time one and time two respectively.

**Table 3:** Paired Samples T-Test for Portion 2 Test-Retest

**Trial Pair Time 1 and Time 2**	**95% Lower Confidence Interval**	**95% Upper Confidence Interval**	**Significance (2 tailed)**
Trial 1	-.02	.78	0.06
Trial 2	.10	1.03	0.02
Trial 3	.12	1.17	0.02
Trial 4	.10	1.17	0.02
Trial 5	.13	1.16	0.01
Trial 6	.02	1.13	0.04
Trial 7	.09	1.17	0.02

The mean absolute error for all subjects for each trial 1–20 can be found in Figure 3. The graphical analysis of the 20 trials suggests over-sampling. The graph exhibits that during trials 1–7, performance plateaued, while during trials 8–20, reposition sense error increased. Trials 1–3 indicated the trials required to improve performance consistency and trials 4–7 were the most consistent trials.

Reposition sense error for all 20 trials was broken into subgroups of four trials. Linear regression results for five of the four trial groups (4–7, 8–11, 15–18, 16–19, and 17–20) identified β coefficients for the slope of the regression line that were close to zero. Any one of these five sets of 4 trials could be considered the appropriate number of trials to perform with the device. The mean absolute repositioning error for group 4–7 was 2.26 degrees, the lowest value, while values for the other four non-significant groups ranged from 2.49 (trials 8–11), 2.98 (trials 15–18), 2.99 (trials 16–19), and 3.06 (trials 17–20) degrees respectively [73]. These results substantiated using seven trials in subsequent reliability studies (portion 2) in particular using trials 1–3 as practice trials and trials 4–7, as the test.

### Portion 2: Test-retest reliability

Trials 2–7 from the paired samples t-test results were statistically significant (Table 3). Consistent differences were found between time 1 and time 2 across all seven trials except for the first trial. Knowing that trials 4–7 produce the best reproducibility, seven trials were performed by the subjects for the test-retest portion. Comparison of all combinations of the seven trials (using a minimum of two and up to seven trials) produced all low ICC (3, k) values with greater values for trials 4–7. Trials 4–7 were chosen for the test-retest portion because the smallest reposition sense error occurred over these trials in the initial repeated trials portion. Subjects tested on two occasions one week apart demonstrated ICC (3,1) values for trials 4–7 of 0.79 (95% CI, 0.71, 0.86; SEM 0.28°) and 0.76 (95% CI, 0.67, 0.84; SEM 0.40°), time one and time two, respectively. These ICCs are indicative of good reliability [70] with low SEM.

**Figure 4:**
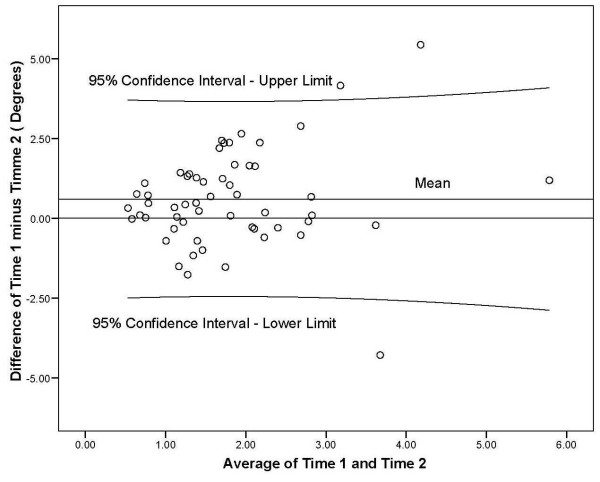
The Bland Altman plot comparing time one and time two for test-retest reposition mean error degree measures with mean and 95% confidence interval.

Using the average value from trials 4 to 7 for time one and time two, an ICC (3,k) of 0.38 (95% CI, -0.06, 0.63; SEM 3.32°) was found for test-retest reliability. This ICC is indicative of poor to moderate reliability [70]. The Bland-Altman method showed all of the measurements except three falling within the 95% confidence limits (Figure 4). The differences are close to zero suggesting both testing times are producing the same results.

### Portion 3: Validity

**Figure 5:**
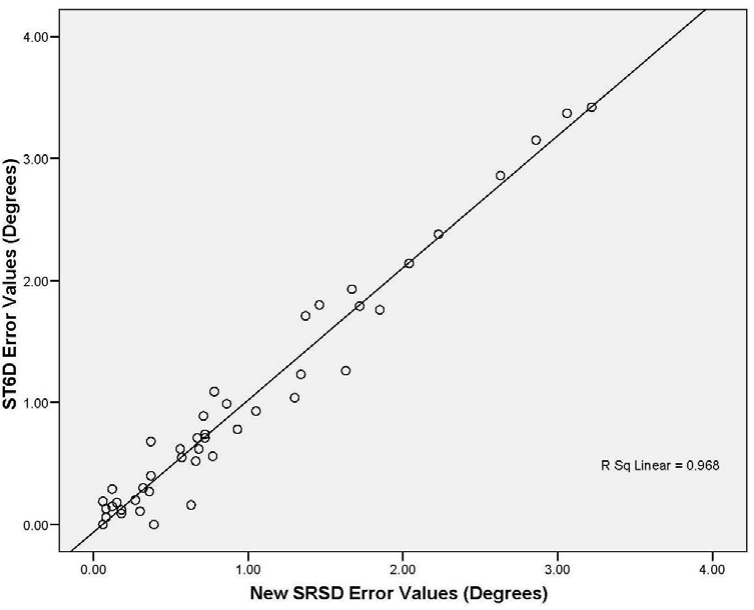
A plot of line of equality for reposition values comparing the ST6D and the new reposition sense device (degree measurements).

**Figure 6:**
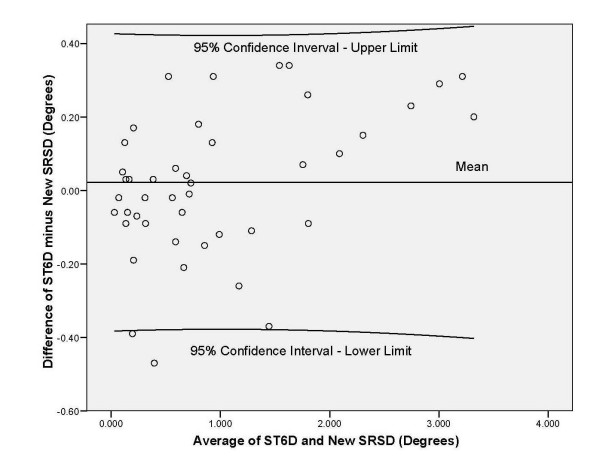
The Bland Altman plot comparing the ST6D to the new reposition sense device (degree measurements) with mean and 95% confidence interval.

Comparing angles computed from the displacement data from the ST6D system and the new SRSD produced an ICC (3,1) of 0.99 (CI 0.55, 0.99; SEM 0.47). The plot of the ST6D measures against the new SRSD (Figure 5) for the single subject measurements indicated both techniques gave similar readings each time as indicated by the line of equality. The Bland Altman plot (Figure 6) showed the mean difference (0.020 degrees) between the measurement techniques and the range in which 95% of the differences lie. Most measures except two lie within the 95% confidence range which suggested a normal distribution. The difference between the two techniques (limits of agreement) was ± 0.40 degrees. These error values fall within values documented in the literature [1,11,38,49]. Also the average of the differences was close to zero suggesting both techniques were producing the same results [70].

## Discussion

### Portion 1: Trunk reposition sense error

The graphical analysis and the use of linear regression indicated the use of trials 1–7 for further testing. According to previous literature, the range of mean ARE for flexion movements of the trunk was from 1.67 – 6.53° [1,11,38,49]. In this study, the mean absolute repositioning error range for all 20 trials was 1.84 – 2.68°. These findings (< 3° on figure 3) are consistent with what has been reported in the literature.

Increasing error values over repeated trials may be an indication of fatigue [48,52-61]. Graphical analysis supported that subject performance declined over trials. In addition, the increase in mean ARE over trials suggested declining reposition sense. We hypothesized that peripheral and/or central fatigue [56,57,73] may have contributed to this decrease in performance. Future studies should examine this trend using electromyographic analysis or near-infrared spectroscopy [74] in an attempt to confirm the effect of fatigue on reposition sense performance.

### Portion 2: Test-retest reliability

The significant findings between time 1 and time 2 for trials 2–7 (Table 3) indicated systematic changes between the test and retest findings. Poor to moderate repeatability ICC (3,k) for trials 4–7 (0.38) were found for test-retest reliability. Similar low ICC test-retest values have been found in the literature at the spine. Swinkels & Dolan [1] reported day to day reliability for lumbar flexion ranging from 0.57 to 0.72 (single factor ANOVA). Koumantakis et al [49] reported ICC (3,3) for lumbar flexion for controls and patients with low back pain of 0.45 (0.96°) and 0.53 (1.25°) with SEM values. Brumagne et al [45] indicated an ICC (1,1) of 0.51 with SEM values for day 1 and 2 of 0.59° and 0.41° for pelvic repositioning. The SEM values indicated better test stability than the ICC value. Brumagne et al. [46] found an ICC of 0.72 using a one-way ANOVA for pelvic repositioning. Cervical test-retest values from Kristjansson et al. [75] using an ICC (2,1) were from 0.35 to 0.90. These authors [75] found significantly more accurate kinesthetic testing with relocation of common cervical postures versus relocation of uncommon cervical postures. Because of the discrepancies in ICC values and plots of data (Bland-Altman method) [72], the use of ICCs as the only measure of reliability was questioned. Results from the above studies suggest that reliability of repeated measurements cannot be evaluated by correlation coefficients alone. The SEM and/or the Bland Altman 95% limits of agreement should be used to interpret the magnitude of disagreement between measures [76,77]. Our low ICC (3,k) 0.38 may be of less concern due to the SEM (3.32°) suggesting that the measurement inconsistency is occurring in an acceptable range or as evidenced in the Bland Altman plot that the repeated testing times are producing similar values.

The poor test-retest ICC values in the present study and previous studies are probably reflective of the increased number of joints involved in producing spinal movement. Greater errors have been produced in the spine than at the extremity joints reflecting spine complexity [78-81]. Also memory becomes important when subjects are expected to reproduce the two-third's full flexion position expected within the test-retest portion of this study one week later. Kristjansson et al. [75] found accuracy was better when common postures were reproduced. Subjects were not oriented or trained to the two-third's full flexion position.

Comparison of the subject's mean full flexion position value to the two-thirds position at time one and time two, indicated the subjects were producing a two-thirds position (see Table 4). Memory and/or motor control issues may impact the differences in testing from time one and time two. The good ICC (3,1) for time one and time two of 0.79 and 0.76 respectively and the very low SEM values (0.28 – 0.40 degrees, respectively) suggested subjects can reproduce a two-thirds position reliably but may have problems replicating those same positions in a retest situation. These test-retest reliability concerns will need to be considered when the device is used throughout a client's extended physical therapy program.

**Table 4:** Mean Degrees ± Standard Deviation for Neutral, Full Flexion and the Two-Thirds (2/3) Flexion Angular Measures for Test (Time One) and Retest (Time Two)

**Test: Time One**				**Retest: Time Two**			
Neutral	Full Flexion	Two-Thirds Flexion	Percentage of Full Flexion	Neutral	Full Flexion	Two-Thirds Flexion	Percentage of Full Flexion

12.17 ± 1.75	47.93 ± 6.43	35.95 ± 4.54	66.5	12.67 ± 1.88	48.15 ± 6.65	36.64 ± 4.93	67.6

### Portion 3: Validity

The ICC findings for comparison of the displacement measures from the ST6D system and the new SRSD suggested excellent agreement of the two techniques using displacement measures. The Bland Altman technique allowed determination of how well the new spine reposition sense device agreed with the gold standard measurement. The findings indicated the new SRSD method has similar reliability compared to the ST6D technique. The Bland Altman technique allowed determination of how well the new reposition sense device agreed with the gold standard measurement. Our findings indicated the new reposition sense method has the same degree of accuracy as the ST6D technique in the sagittal plane. The new SRSD's methodology is valid.

### Clinical relevance

Clinicians are currently prescribing proprioceptive retraining programs for patients with back problems [82-86], with justification for carrying out these programs largely based on clinical theory and from proprioception literature addressing peripheral joints. Presently spinal proprioception has not being assessed clinically other than indirectly through balance. Because proprioception impairment may be part of the multifactorial nature of spinal pain it should be evaluated and various intervention strategies should be assessed to determine their efficaciousness [87-89]. Sagittal plane reposition sense can be reliably assessed using this new SRSD. Various types of intervention programs, used to treat patients with spinal dysfunction, could be examined for their effectiveness in improving sagittal plane reposition sense by evaluation with this new device. By improving proprioception in patients with low back pain, dysfunction may improve as has been found in the peripheral joints.

### Future studies

The new SRSD needs to be evaluated with people with chronic disease or chronic low back pain to assess reliability within these populations.

## Conclusion

The repeated trials, test-retest and validity testing against the ST6D system provided evidence supporting the use of the new SRSD to measure sagittal trunk reposition sense. This work demonstrated reposition sense performance decreasing over 20 trials, indicating the use of 7 trials and specifically trials 4–7 for data analysis. The mean absolute repositioning error range during the repeated trials portion was 1.84 – 2.68°, falling within the previously reported range of values in the literature. Comparison of the device to the ST6D system indicated comparable measures to allow the new SRSD to be used in the sagittal plane in place of the gold standard ST6D system.

## Competing interests

The author(s) declare that they have no competing interests.

## Authors' contributions

All authors contributed equally to this work and read and approved the final manuscript. Ms. Bulow and Ms. Ewers-Panveno were students in the Department of Physical Therapy and Human Movement Sciences, Northwestern University Medical School, under the supervision of Ms. Petersen, at the time when the repeated trials phase 1 portion was conducted, as part of the DPT requirement.
